# Scale‐Dependent Attraction of Invasive Raccoons to Bait Sites: Behavioural and Proximity Responses in a Post‐Disaster Agricultural Landscape

**DOI:** 10.1002/ece3.73722

**Published:** 2026-06-02

**Authors:** Akira Watanabe, Daisuke Nakamura, Masataka Yoshida, Norihiro Hoshi

**Affiliations:** ^1^ Hama Agricultural Regeneration Research Centre Fukushima Agricultural Technology Centre Minamisoma City Fukushima Prefecture Japan; ^2^ Institute of Livestock and Grassland Science National Agriculture and Food Research Organization (NARO) Tsukuba City Ibaraki Prefecture Japan; ^3^ Rural Areas Development Division Department of Agriculture, Forestry and Fisheries Fukushima City Fukushima Prefecture Japan; ^4^ Tohoku Agricultural Research Center National Agriculture and Food Research Organization (NARO) Fukushima City Fukushima Prefecture Japan

**Keywords:** baiting, cafeteria experiment, daily minimum distance, hidden Markov model, raccoon

## Abstract

Baiting is widely used to improve the capture and observation of invasive mesocarnivores, but it can also alter behaviour and distribution, potentially increasing ecological risks, such as non‐target interactions and pathogen transmission. However, responses to baiting across temporal scales remain poorly understood. We investigated scale‐dependent responses of invasive raccoons ( *Procyon lotor* ) to baiting in a post‐nuclear‐disaster agricultural landscape in Fukushima, Japan. We used cafeteria‐style bait trials, in which multiple bait types were offered simultaneously at each station; together with GPS telemetry, we evaluated responses from bait choice to short‐, mid‐ (daily), and long‐term space use. Individual bait preferences, short‐term movement responses, daily responses and long‐term space use were analysed using Plackett–Luce models, hidden Markov models, minimum distance to trap sites and autocorrelated kernel density estimation, respectively. Seven GPS‐collared raccoons visited bait stations, yielding 15,837 GPS locations. Bait choice varied markedly among individuals. Across trials, dry cat food (0.40) and caramel corn (0.38) had the highest first‐selection probabilities, whereas dried ramen noodles were seldom selected first (0.01). Some individuals consistently preferred other bait types such as sake lees. Short‐term analyses showed strong attraction to bait stations, with area‐restricted search‐like movements increasing from 22.2% during non‐baiting periods to 53.8% during baiting. At the daily scale, raccoons moved closer to trap sites on baiting days, indicating increased proximity to management locations. In contrast, all bait stations fell within long‐term home ranges and often within core areas, suggesting localised activity intensification rather than broader restructuring of space use. Overall, baiting induced strong but scale‐dependent behavioural responses, with localised increases in site use but little evidence of broader restructuring of long‐term space use. These findings suggest that the effectiveness and interpretation of bait‐based monitoring and management may depend on both temporal and individual‐specific bait preferences.

## Graphical abstract

Using cafeteria‐style bait trials and GPS telemetry, we investigated scale‐dependent responses of invasive raccoons to baiting in a post‐nuclear‐disaster agricultural landscape in Fukushima, Japan. Baiting induced strong short‐term and daily‐scale attraction to trap sites but did not restructure long‐term space use, highlighting the need to balance capture efficiency with potential ecological risks.

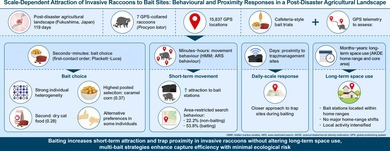

## 1 Introduction

Biological invasion is a major socio‐ecological challenge involving the increase of invasive species, including invasive mesocarnivores, and alters ecosystem functioning while reducing biodiversity globally (Clavero and García‐Berthou 2005; Mack et al. 2000; Strayer 2012). In landscapes disturbed by anthropogenic activities or natural disasters, changes in resource availability and management pressure can facilitate the establishment and geographical expansion of non‐native species (Didham et al. 2007; Hobbs and Huenneke 1992). Specifically, the cessation of human management practices has been identified as a key driver of invasion processes in such landscapes (Hirose et al. 2021).

The raccoon ( *Procyon lotor* ) is a highly adaptable and omnivorous invasive mesocarnivore that occupies diverse environments, including urban and rural landscapes (Fischer et al. 2017; García et al. 2012; Salgado 2018). In Japan, feral raccoons were first documented in 1962. Their rapid population growth followed increased importation as pets in the late 1970s, with subsequent abandonment or intentional release, or by escape (Doi et al. 2018; Ikeda 2006). Government records further indicate that since the 1970s, raccoon capture has expanded nationwide, reflecting the widespread establishment of this species, with numerous localities designating it as a target species for control.

Following the 2011 Fukushima Daiichi Nuclear Power Plant accident, human activities abruptly and extensively ceased within evacuation zones and surrounding areas, resulting in the rapid emergence of unmanaged farmland, abandoned residential areas and dense vegetation. This rapid ‘human abandonment’ has also positioned Fukushima as a unique socio‐ecological setting for examining wildlife responses to sudden changes in human presence (Lyons et al. 2020). Camera‐trap studies report marked changes in the populations of large and medium‐sized mammals within and outside the evacuation zones (Fukasawa et al. 2016). Prefectural management reports indicate that the raccoon population was limited in the mid‐2000s but expanded rapidly across numerous municipalities during the 2010s after the tsunami and nuclear accident. Reports of house intrusion, faecal contamination, crop damage and predation on threatened amphibians further highlight the urgency of invasive species management in post‐disaster recovery regions (Fukushima Prefecture 2021).

In depopulated post‐disaster regions, wildlife management often faces constraints in personnel, operational funding and long‐term monitoring capacity; therefore, baiting, which alters animal behaviour, contact structure and distribution, is used to enhance capture efficiency and increase trap‐site visitation rates for invasive mesocarnivores. However, inappropriate baiting practices may result in non‐target consumption and the aggregation of individuals, increasing disease transmission risk (Sorensen et al. 2014). Moreover, the effectiveness of baiting as a management intervention strongly depends on design factors, including bait density, spatial arrangement and season and habitat contexts (Hill et al. 2024; Sattler et al. 2009). In addition, competition with non‐target species can constrain management outcomes (Olson et al. 2000; Pedersen et al. 2019). Although baiting can improve short‐term capture, it can also have undesirable long‐term consequences, including repeated use of baited areas, localised concentration of activity around bait sites, or increased movement by remaining individuals following intervention (Snow and VerCauteren 2019).

Most management‐oriented studies focus on operational metrics such as capture rate or visitation frequency, including comparisons of trap success, bait uptake and site visitation under different bait types or deployment strategies (Hill et al. 2024; Olson et al. 2000; Sattler et al. 2009), rather than explicitly testing how baiting alters animal movement and space use. Thus, the mechanisms underlying bait‐driven responses remain unclear, particularly in terms of distribution across multiple temporal scales. Studies investigating whether baiting only induces short‐term behavioural attraction (e.g., temporary residency or area‐restricted movements near bait sites) or induces measurable changes in spatial proximity to management sites over longer temporal windows relevant to capture operations are limited. Therefore, clarifying these processes is important, as short‐term attraction may enhance trapping efficiency but sustained local concentration may elevate non‐target interactions and disease transmission risk (Snow and VerCauteren 2019; Sorensen et al. 2014).

In this study, we evaluated the responses of raccoons to baiting across multiple temporal scales in a post‐nuclear‐disaster agricultural landscape in Fukushima, Japan. Employing commercially available baits, we conducted a cafeteria‐style feeding experiment in which several bait types were offered simultaneously within the same trial to quantify individual bait preferences using Plackett–Luce models. Then, to examine movement responses to baiting at short and intermediate time scales, we used GPS telemetry. Specifically, short‐term behavioural attraction was assessed using hidden Markov models (HMMs), testing whether baiting and distance to bait sites influenced transition into area‐restricted search behaviour (Langrock et al. 2012; McClintock et al. 2020). To quantify mid‐term proximity effects relevant to capture operations, we analysed daily minimum distances to trap sites during baiting and non‐baiting periods. Finally, we estimated longer‐term space‐use patterns using autocorrelated kernel density estimation (AKDE), which accounts for temporal autocorrelation and irregular sampling (Calabrese et al. 2016; Fleming et al. 2015). Our findings may reveal how baiting shapes invasive raccoon behaviour and spatial proximity to management sites over both short and long time scales.

## 2 Materials and Methods

**FIGURE 1:**
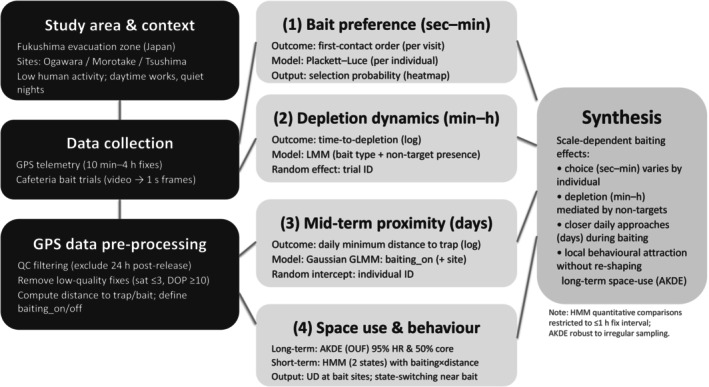
Overview of the study workflow and analytical framework. Abbreviations: AKDE, autocorrelated kernel density estimation; DOP, dilution of precision; GLMM, generalised linear mixed model; HMM, hidden Markov model; HR, hazard ratio; LMM, linear mixed model; OUF, Ornstein–Uhlenbeck foraging; UD, utilisation distribution.

To evaluate raccoon responses to baiting across multiple temporal scales, we combined feeding‐trial observations, camera‐trap records and GPS telemetry within a unified analytical framework (Figure 1).

### 2.1 Study Area

**FIGURE 2:**
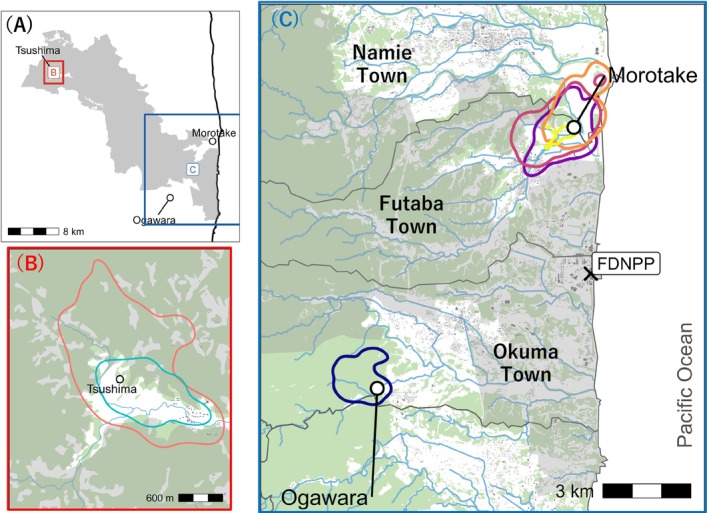
Map of the study area in Fukushima Prefecture, Japan. (A) Overview of the three bait‐site areas within the difficult‐to‐return zone; rectangles indicate the areas enlarged in panels B and C. (B) Close‐up of the Tsushima bait‐site area. (C) Close‐up of the Morotake and Ogawara bait‐site areas, showing their proximity to the Fukushima Daiichi Nuclear Power Plant (FDNPP) and the Pacific Ocean. Grey shading indicates the difficult‐to‐return zone, excluding Specified Reconstruction and Revitalization Base Areas where evacuation orders had been lifted. Coloured outlines show individual 95% AKDE home‐range isopleths, white circles indicate bait sites, green shading indicates forest cover, blue lines indicate streams, grey features indicate residential structures, and thin dark lines indicate municipal boundaries.

The study was conducted at three sites within the Fukushima Prefecture evacuation zones (Ogawara, Morotake and Tsushima, located in Okuma, Futaba and Namie Towns, respectively) established following the Fukushima Daiichi Nuclear Power Plant accident of March 2011 in Japan (Figure 2). Since 2011, these areas have experienced prolonged declines in human activity, resulting in a post‐disaster landscape characterised by unmanaged farmlands, abandoned residential areas and regenerated vegetation. Regeneration occurred mainly in abandoned paddy fields and surrounding lowland habitats, where cessation of cultivation led to rapid drying and the spread of spontaneous herbaceous vegetation (Lyons et al. 2020; Matsushima et al. 2021). The area has a temperate climate, with relatively little snowfall and mean annual temperature and precipitation of 14.7°C and 1205 mm, respectively, based on averages from the Namie Meteorological Station for the study period (Japan Meteorological Agency 2026). Human presence remained limited during the study period and in municipalities encompassing the study area, resident return remained well below pre‐disaster levels; official prefectural summaries indicate that Okuma, Futaba and Namie were among the municipalities where resident populations remained below half of pre‐disaster levels as of 2024. Agriculture has also only partially resumed. The proportion of farmland where farming had resumed was 4.2%, 0.5% and 26.8% in Okuma, Futaba and Namie, respectively, by the end of fiscal year 2024, which is well below the 49.7% average across the 12 municipalities affected by evacuation orders (Fukushima Prefecture 2024). Based on field observations during the study period, roads in the area were used frequently by construction vehicles during the daytime for recovery and decontamination work, whereas nighttime human activity was generally low, creating a marked diel contrast in anthropogenic disturbance that may favour nocturnal wildlife movement. Within the study region, decontamination was prioritised in the three study sites relative to other evacuation areas. Evacuation orders were lifted in March 2020 in Tsushima and Morotake, and in April 2023 in Ogawara (Fukushima Prefecture 2026). Prior to the nuclear accident, the landscape comprised mainly residential areas interspersed with rice paddies.

### 2.2 Study Design and Analytical Workflow Overview

Our study evaluated raccoon responses to baiting across four temporal scales. We assessed (1) bait preference at feeding stations from first‐contact order during cafeteria‐style trials (seconds to minutes), (2) bait depletion dynamics and potential non‐target interference assessed using video‐recorded consumption events (minutes to hours), (3) mid‐term spatial proximity to bait sites using daily minimum distances based on GPS tracking (1‐day scale) and (4) short‐ and long‐term movement and space use using HMMs (minutes to hours) and AKDE to estimate seasonal‐scale space use (weeks to months). Temporal scales were defined a priori based on ecological relevance and data resolution. GPS relocation data were filtered for quality and to minimise capture‐related behavioural artefacts before analysis.

### 2.3 Raccoon Capture and GPS Tagging

Raccoons were live‐captured between June 2023 and August 2025 using five medium‐sized cage traps with a treadle mechanism (Havahart, Woodstream Corp., Lititz, PA, USA) or a modified Rakutory trap (Sarji Miyawaki Co. Ltd., Osaka, Japan). At all trap sites, cat food (Cannet Chip Fish, Petline Co. Ltd., Tajimi, Japan) was used as bait, and traps were checked each morning. The same product was later used as the dry‐cat‐food bait in the cafeteria‐style feeding trials, where it served as an animal‐based bait type in contrast to caramel corn. Capture locations were recorded for all GPS‐collared raccoons, and the distance from each capture site to the nearest bait station is presented in Table 1. For the seven individuals retained for bait‐response analyses, these distances ranged from 3 to 1151 m, indicating that some individuals were captured at or very close to bait stations whereas others were captured substantially farther away. The captured individuals were immobilised on the day of capture via an intramuscular injection of ketamine hydrochloride (Ketalar, 10 mg kg^−1^; Pfizer Inc., New York, NY, USA) and medetomidine hydrochloride (Domitor, 0.08 mg kg^−1^; Zoetis Inc., Kalamazoo, MI, USA). Thereafter, the individuals were weighed using an electronic scale, and standard external morphometric measurements were taken. GPS collars were then fitted to each individual, ensuring that the mass of the collar did not exceed 5% of their body weight. Anaesthesia was reversed with an intramuscular injection of the antagonist atipamezole hydrochloride, after which animals were allowed to recover and then released. The GPS collars used were LiteTrack series models (Lotek Wireless Inc., Newmarket, ON, Canada), including LiteTrack 150 Iridium, LiteTrack 140 RF and LiteTrack 60 RF. Furthermore, to enable individual identification in nocturnal black‐and‐white images, we marked collars with distinctive vinyl tape patterns. All capture and handling procedures were approved by the Animal Care and Use Committee of the National Agriculture and Food Research Organisation (permit no. 21B172ILGS‐3).

**TABLE 1:** Individual‐specific long‐term home‐range and core‐area sizes obtained via autocorrelated kernel density estimation (AKDE).

Individual	No. of fixes	Tracking period	Fix interval	95% home range (km^2^)	50% core area (km^2^)	Distance from capture site to nearest bait station (m)	No. of feeding visits
Rac01F	296	30 Jun 2023–12 Dec 2023	4 h	8.41	1.87	12	7
Rac02M	352	20 Oct 2023–8 Apr 2024	4 h	13.41	3.25	1151	24
Rac03M	5458	25 Feb 2024–17 Jul 2024	Mixed^a^	16.72	4.39	1151	15
Rac04M	2168	24 Apr 2024–15 May 2024	10 min	12.96	2.10	3	3
Rac05F	1813	23 May 2024–19 Jul 2024	30 min	0.98	0.11	1151	1
Rac06M	5267	21 Oct 2024–31 Jul 2025	30 min	9.36	2.17	357	5
Rac07F	390	7 Aug 2025–7 Oct 2025	1 h	2.91	0.60	5	2

*Note:* Home‐range area (95% utilisation distribution isopleth) and core‐area size (50% isopleth) are reported as point estimates (km^2^). *n* represents the number of retained GPS fixes per individual after quality filtering.

^a^ Rac03M was tracked at 4‐h intervals from 25 Feb 2024 to 25 Apr 2024 and at 10‐min intervals from 25 Apr 2024 to 17 Jul 2024.

### 2.4 Collection of Tracking Data and Fix Schedules

The GPS collars were programmed to record data at variable fix intervals to balance long‐term monitoring with battery conservation and to accommodate logistical constraints typical of field‐based telemetry studies. Fix intervals varied between individuals and, in some cases, within individuals over time, ranging from 10 min to 4 h. Shorter intervals were implemented during targeted periods when fine‐scale movement data were required (e.g., during intensified monitoring to capture bait‐site visits). Longer intervals were used during earlier deployments to prolong collar life and maximise tracking duration. Specifically, two individuals (one female and one male) were initially monitored at 4‐h intervals during early deployments (June 2023 to February 2024). Subsequently in April 2024, one male was recaptured and its collar was reprogrammed to a 10‐min fix interval; thereafter, three other males were monitored at 10‐min intervals. One individual did not visit bait sites during the monitoring period, and thus, provided no bait‐interaction data; accordingly, data from this individual were not included in bait‐related behavioural analyses. This exclusion was methodological rather than outcome‐driven, as the individual still contributed to movement and space‐use analyses where bait visitation was not a prerequisite. During the final deployment period (August 2025), two individuals were monitored at 30‐min intervals and one female was monitored at 1‐h intervals. Overall, GPS data were obtained from nine individuals (six males and three females). The resulting data were characterised by heterogeneous temporal resolutions across individuals and periods. However, this issue was explicitly accommodated in subsequent analyses via a continuous‐time movement modelling framework and AKDE, which are robust to irregular sampling schedules and allow consistent inference across tracking regimes (Calabrese et al. 2016; Fleming et al. 2015).

### 2.5 GPS Data Filtering and Quality Control

To ensure the accuracy and biological relevance of the recorded GPS data, location data recorded within 24 h following anaesthetic antagonist administration were excluded as post‐capture movement and space use typically deviate from baseline patterns (Cagnacci et al. 2010; Hebblewhite and Haydon 2010; Morellet et al. 2009). This timing was defined relative to antagonist administration to represent voluntary movement onset following immobilisation. Thus, the effects of capture‐ and handling‐related behavioural artefacts were minimised. Location data acquired from approximately three satellites or with dilution of precision values ≥ 10 were excluded to eliminate the effects of elevated positional error (D'eon and Delparte 2005; Frair et al. 2010; Lewis et al. 2007). Data for locations during the night of recapture were also excluded to avoid capture‐related disturbance. These filtering steps were applied consistently across individuals and deployment periods to improve the reliability of subsequent space‐use and movement analyses.

### 2.6 Selective Feeding Experiment

**FIGURE 3:**
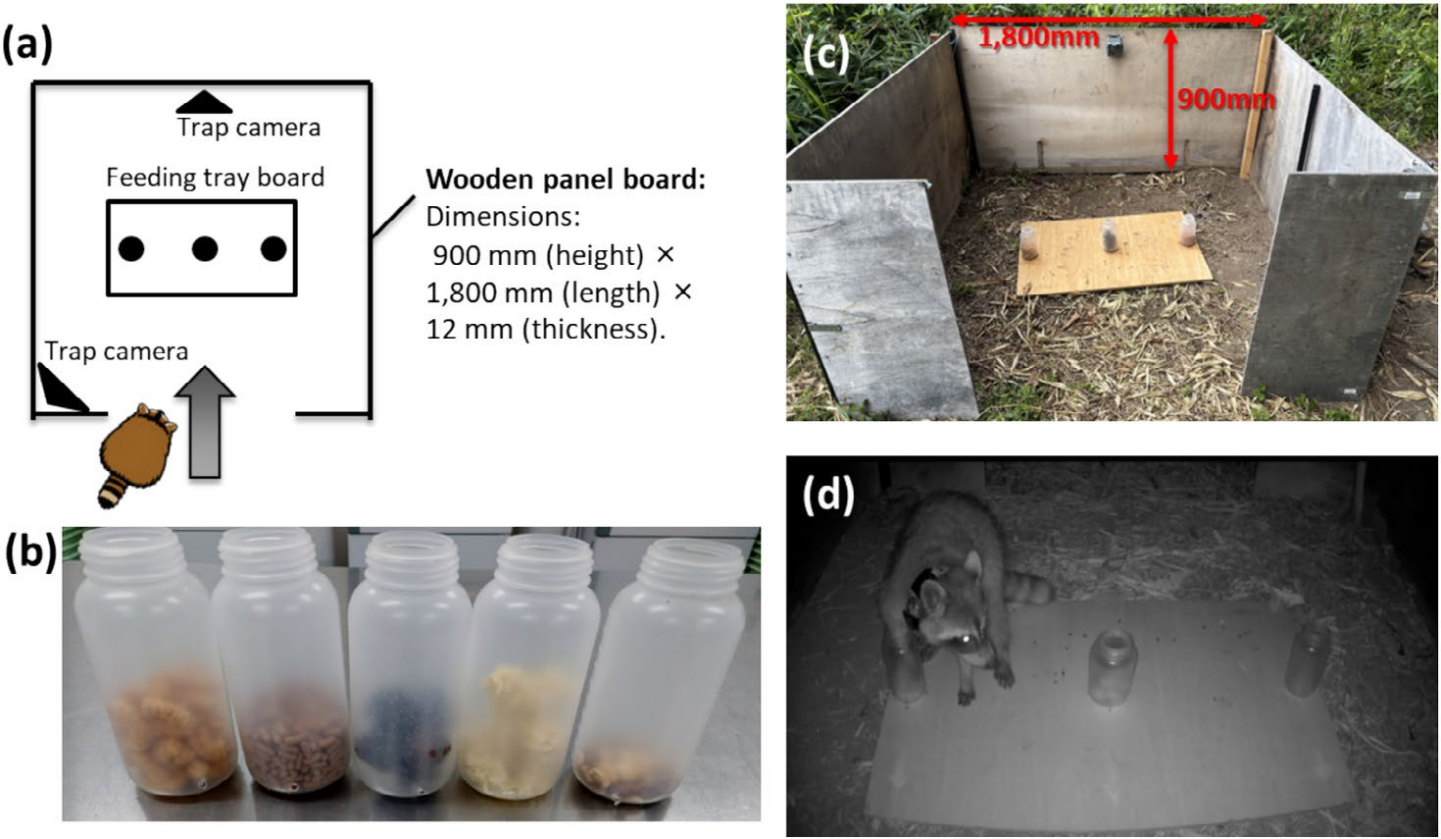
(a) Schematic representation of the feeding station. Bait containers were fixed to a wooden board panel with a single entry point (arrow) ensuring that each raccoon viewed all bait containers from an approximately equal distance. (b) Plastic bait containers (opening diameter, 42 mm; height, 162 mm). From left to right, the containers shown in the photograph contain Tohato Caramel Corn, dry cat food, sake lees, dry ramen noodles and Japanese mixed nuts. All five bait types used in the study are shown for reference, but only three bait types were presented simultaneously in any given trial: Caramel corn, dry cat food and one alternative bait. (c) Oblique view of a feeding station at the Tsushima site, showing the enclosure structure and the vertical and horizontal dimensions of the setup (1800 mm × 900 mm). (d) Example of a raccoon (Rac06M) interacting with the bait containers, recorded by an infrared camera during a feeding trial.

At the study sites and close to bait stations, non‐target mesocarnivores and other wildlife, including raccoon dogs (*Nyctereutes viverrinus*), wild boar ( *Sus scrofa* ) and masked palm civets ( *Paguma larvata* ), as well as birds and small rodents, were regularly recorded by infrared camera traps throughout the experimental period. This community context was relevant given that non‐target interference and bait removal can strongly influence baiting outcomes and trap effectiveness. Raccoon bait preference was assessed using a multi‐choice “cafeteria‐style” feeding experiment conducted at three study sites. One bait station was established at each site, giving a total of three bait stations. At each station, three bait types were presented simultaneously during each trial. The trials were conducted over a total of 119 days between 6 December 2023 and 6 June 2024, and between 17 July and 25 August 2025. We defined one trial as a single bait presentation event at a station and conducted 57 trials in total. Two commercially available baits frequently used in raccoon control programmes in Japan, caramel corn and dry cat food, were used as standard reference baits. A third bait was selected from one of three alternatives (mixed nuts, dried ramen noodles, or sake lees). Accordingly, each trial included three bait types presented simultaneously: two reference baits and one alternative bait. Each alternative bait was evaluated over a predefined number of trials (dried ramen noodles: 15 trials; mixed nuts: 13 trials; sake lees: 29 trials), and alternatives that consistently showed a lower selection rate than the reference baits were subsequently replaced by the next candidate. In each trial, all baits were provided in equal amounts (30 g) and replaced every 2 days during the experimental periods. All baits were presented simultaneously and were fully visible from a single vantage point, minimising sequential choice effects (Meier et al. 2012). To reduce potential bias arising from approach direction, access routes to each feeding station were restricted. The baits were placed within a wooden enclosure (1.8 m × 0.9 m) with a single entrance opening (Figure 3).

During pilot trials, non‐target species, such as raccoon dogs and corvids (*Corvus* spp.), frequently consumed the baits before raccoons arrived. To reduce non‐target access, a semi‐transparent plastic container (entrance diameter, 4.2 cm; height, 16.2 cm) was fixed to the wooden board at each bait position. The entrance dimensions, comparable to those of commercially available egg‐type traps, are applicable in wildlife management contexts.

Animal visits and feeding behaviour were recorded using two infrared motion‐triggered camera traps (Hyke SP3; Hyke Co. Ltd., Sendai, Japan). The cameras were programmed to record 60‐s videos without a trigger interval between consecutive recordings. One camera was positioned directly in front of the entrance of the bait site to capture individual identity and entry behaviour, whereas the second camera was positioned to minimise blind spots caused by shadows. Feeding trials were suspended on rainy days as the dry baits were sensitive to rainfall.

The recorded videos were converted to still images at a one‐frame‐per‐second rate. Thereafter, the images were annotated using Timelapse (version 2.3.2.8; Greenberg et al. 2019). The annotation included: (i) species identity, including individual identification of collared raccoons; (ii) proximity of each individual to the three bait types; and (iii) feeding‐related actions, defined as placing the nose at the container entrance or inserting a forelimb into the bait container. Observations from both cameras were used to obtain animal behaviour at a 1‐s resolution. Then, to effectively screen images without animal presence, the object‐detection and bounding‐box functions in Timelapse were used.

### 2.7 Analyses

#### 2.7.1 Order of Bait Selection

Bait preference was assessed using a cafeteria‐style design in which multiple bait types were presented simultaneously. Thus, ranked‐choice data were obtained and subsequently analysed using Plackett–Luce models using the ‘PlackettLuce’ package in R (Turner et al. 2020). These models estimates relative selection probabilities from incomplete and tied rankings. We inferred preference from the order of selection rather than absolute consumption, identifying bait types most likely to be selected first. This approach was suitable for our experimental design, where the number and combination of bait types varied among trials, with some baits not contacted, resulting in partial or incomplete rankings. Furthermore, contact with multiple baits sometimes occurred within a short time window, making it difficult to unambiguously determine first contact. In cases where multiple baits were contacted within 5 s, contacts were treated as tied ranks, andPlackett–Luce models with tie handling were applied. Rankings were constructed from the temporal sequence of first bait contacts within each trial for each identifiable individual, with each feeding visit treated as a single ranking observation. The Plackett–Luce models were fitted separately for each individual as bait preference was expected to differ substantially. Subsequently, estimated relative selection probabilities were visualised using heatmaps to facilitate comparison among individuals and bait types.

#### 2.7.2 Speed of Bait Consumption

To assess differences in bait consumption speed, we estimated the time required for each bait to be depleted based on the interval between the start of feeding and the final observed feeding event for a given bait type. Camera footage data were also used to identify raccoon dog visits and their duration, as they could affect bait depletion rates. Consumption time was modelled as the response variable in a linear mixed‐effects model, with bait type and raccoon dog presence as fixed effects. Trial identity was also included as a random effect to account for among‐trial variation in overall attractiveness or ‘sink effects’ (i.e., differential site‐level bait removal unrelated to bait type). Consumption times were further log‐transformed to account for right‐skewed distributions. The necessity of including trial as a random effect was also evaluated using likelihood ratio tests.

#### 2.7.3 Long‐ and Short‐Term Effects of Baiting on Raccoon Space Use and Behaviour

##### 2.7.3.1 Long‐Term Space Use

Long‐term space use was quantified via AKDE, which accounts for temporal autocorrelation and irregular sampling in animal movement data (Fleming et al. 2015). All analyses were performed using the ‘ctmm’ package in R (Calabrese et al. 2016). For each individual, movement trajectories were modelled using continuous‐time stochastic movement models, and Ornstein–Uhlenbeck Foraging models were fitted using perturbative hybrid restricted maximum likelihood estimation (pHREML), as recommended for AKDE‐based home ‐ range estimation (Silva et al. 2022). This approach explicitly accounts for autocorrelation in both position and velocity, avoiding biases associated with conventional kernel density estimators applied to autocorrelated relocation data (Noonan et al. 2019). Additionally, utilisation distributions (UDs) were estimated via AKDE, and home ranges and core areas were defined based on 95% and 50% isopleths, respectively. Furthermore, to assess the spatial relationship between bait sites and long‐term space use, UD values were extracted at bait‐site location and compared with individual‐specific thresholds corresponding to the 50% and 95% UD isopleths. Given that AKDE is designed for autocorrelated and irregular tracking data, all individuals were retained for long‐term analyses regardless of their fix schedules.

##### 2.7.3.2 Mid‐Term Proximity to Bait Sites (Daily Minimum Distance)

Baiting periods were defined as dates during which cafeteria‐style feeding trials were conducted at each site (‘baiting_on’), whereas all other monitoring dates were classified as non‐baiting periods (‘baiting_off’). One feeding trial was defined as a single bait‐presentation period at one station. Across the study area there were 57 baiting periods in total (Ogawara, 9; Morotake, 37; Tsushima, 11), each lasting 1–3 days. This definition was applied consistently to each individual while considering site assignment. We then quantified the daily minimum distance between each individual and the bait site within the study area to evaluate whether baiting influenced intermediate‐scale proximity to management locations. For each individual‐day, the minimum distance to the bait site was calculated using all GPS fixes recorded on a given day. Daily minimum distance reflects whether an individual approached the bait site at least once a day, with reduced sensitivity to within‐day temporal autocorrelation relative to fix‐level binary proximity indices. The values obtained were log‐transformed prior to modelling given that the daily minimum distances were right‐skewed. First, we tested whether daily minimum distances differed between baiting and non‐baiting periods using a Gaussian generalised linear mixed model, with baiting condition set as a fixed effect and individual identity set as a random intercept. Site was also included as an additional fixed effect in alternative models to account for among‐site differences. The models were fitted using the “glmmTMB” package in R (Brooks et al. 2017).

##### 2.7.3.3 Short‐Term Movement Behaviour

Short‐term movement behaviour was analysed using HMMs fitted to step lengths and turning angles (Langrock et al. 2012). Two behavioural states were considered: State 1, representing area‐restricted search (ARS)‐like or residency behaviour, and State 2, representing directed movement or transit. Specifically, step lengths and turning angles were modelled using gamma and von Mises distributions, respectively; HMMs were implemented using the “momentuHMM” package in R (McClintock and Michelot 2018). The most likely sequence of behavioural states was then reconstructed using the Viterbi algorithm (Langrock et al. 2012). Moreover, to evaluate bait effects on state‐switching dynamics, baiting condition and distance to the nearest bait station were included as covariates for transition probabilities. Thus, it was possible to test whether proximity to the bait site increased or decreased the probability of switching into or out of the residency state. This approach builds on recent developments integrating behavioural state dynamics with habitat‐ or resource‐related covariates (Klappstein et al. 2023; McClintock et al. 2020). Additionally, given the sensitivity of behavioural inference from HMMs to temporal resolution, quantitative comparisons of state‐derived metrics were restricted to the five individuals with fix intervals ≤ 1 h (*n* = 5), whereas long‐term space‐use analysis based on AKDE and analyses of daily minimum distance to the nearest bait station included seven individuals (*n* = 7). Individuals monitored at 4‐h intervals were also retained for the qualitative evaluation of bait‐site visitation.

## 3 Results

### 3.1 Capture Summary and GPS Data Availability

Between June 2023 and August 2025, a total of 40 raccoons were live‐captured, of which 22 (14 males, eight females) were fitted with GPS collars, with collaring restricted by body mass (< 5% rule) and sex‐balance considerations. Data from these collared individuals were screened to identify individuals suitable for bait‐related analyses. Seven collared raccoons (four males, three females) visited the bait sites during the cafeteria‐style feeding trials and were included in the analyses of bait preference and short‐term behavioural responses. The remaining collared individuals were excluded, as their collars failed before the feeding trials owing to battery depletion or loss of contact, or because they did not visit any of the bait sites during the experimental period.

Initially, 18,513 recorded locations were obtained for the seven included individuals, and after filtering, 15,837 GPS locations were retained. The fix intervals ranged from 10 min to 4 h depending on deployment timing and battery management (Table 1), with the following numbers of individuals for each fix schedule: 10‐min, *n* = 1; 30‐min, *n* = 2; 1‐h, *n* = 1; 4‐h, *n* = 2; and mixed 4‐h/10‐min, *n* = 1. Individuals with longer fix intervals accounted for fewer locations, but their data were retained for long‐term space‐use and bait‐site visitation analyses.

### 3.2 Order of Bait Selection

Selective feeding was analysed based on videos recorded using infrared camera traps. The videos were converted to 1‐s resolution still images, yielding 392,091 image frames in total, corresponding to 73 distinct feeding visits across 45 trials. Visit counts were uneven among individuals (1–24 visits; Table 1), reflecting substantial variation in visitation frequency to feeding stations. Subsequent extraction and screening showed raccoons in 105,545 frames, of which 92,140 corresponded to GPS ‐ collard raccoons. Although this facilitated individual‐level analyses, the high proportion of detections involving tagged raccoons may partly reflect the fact that some analysed individuals were captured at or very close to bait stations (Table 1). Non‐target species, predominantly raccoon dogs, were frequently recorded at the feeding stations as they accounted for 157,864 frames. Other non‐target animals included rodents (Order Rodentia), red foxes ( *Vulpes vulpes* ) and birds (Class Aves), accounting for approximately 10,000, 973 and 9338 frames, respectively. These records were used to characterise non‐target visitation and assess the influence of raccoon dogs on bait depletion dynamics. However, they were excluded from the analyses of bait preference based on selection order. Thus, we obtained high‐resolution behavioural data that allowed the precise identification of first bait contact and the temporal sequence of bait selection within trials.

**FIGURE 4:**
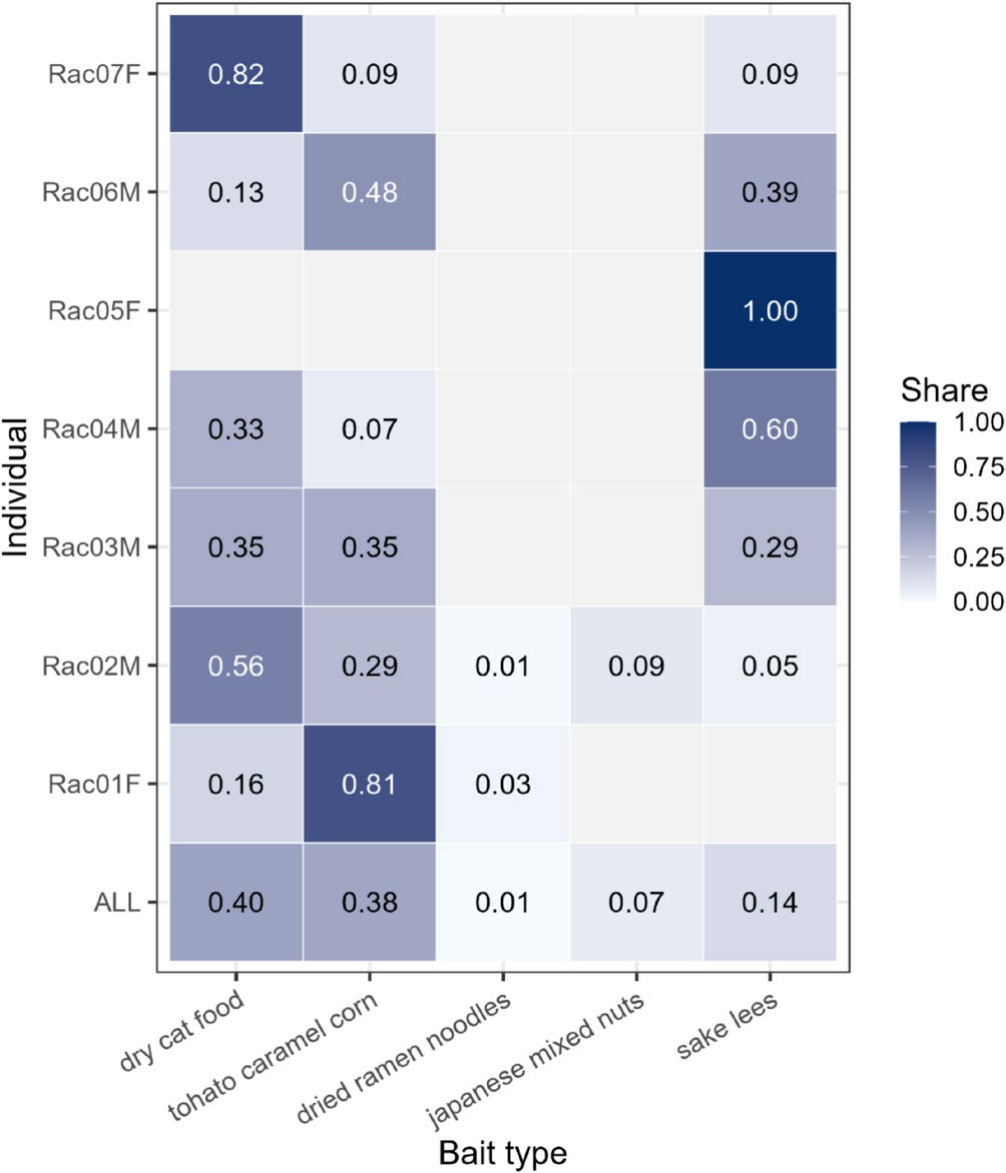
Relative selection probabilities of five bait types estimated using Plackett–Luce models. The cells show the proportion of first contacts attributed to each bait type, based on the order of initial selection (hand or nose contact). The bottom row (‘ALL’) represents pooled estimates across all trials. Subsequent rows show individual‐level estimates, with darker shades indicating higher relative selection probabilities. Bait types not contacted are shown as blank cells.

Plackett–Luce models revealed clear differences in the order of first selection among bait types (Figure 4). When trials were pooled without individual identification, the highest relative selection probability was observed for dry cat food (0.40), followed closely by Tohato caramel corn (0.38), sake lees (0.14), Japanese mixed nuts (0.07) and dried ramen noodles (0.01). Conversely, individual‐level analyses revealed substantial heterogeneity in bait preference (Figure 4). Strong single‐bait preferences were evident in some individuals, whereas others showed more even or mixed first‐selection probabilities across bait types. Across individuals, the dominant first‐selected bait differed, with dry cat food, Tohato caramel corn and sake lees each strongly preferred by different raccoons. However, feeding visit counts varied markedly among individuals (1–24 visits; Table 1), and therefore, the apparent strength of individual preferences should be interpreted with appropriate caution where repeated observations were limited. Overall, these results indicate that bait selection was strongly individual‐specific, and that the effectiveness of baiting may depend not only on bait type but also on behavioural heterogeneity among individuals.

### 3.3 Speed of Bait Consumption

Across the cafeteria‐style feeding trials, 122 bait depletion events were recorded from the 46 trials in which raccoons contacted at least one bait type. In most trials, multiple baits were contacted within a short time window, making the determination of first selection challenging. Additionally, not all bait types were contacted in every trial, resulting in partial or incomplete rankings. First contact was defined as either nose contact or manual manipulation, as confirmed based on infrared camera footage. When first contact occurred nearly simultaneously across two or more bait types, these events were treated as tied selections. Analysis in this regard revealed that bait depletion time differed among the bait types and was influenced by raccoon dogs (Table 2). After log transformation, caramel corn was depleted significantly faster than dried ramen noodles, the reference category in the model, showing the largest negative effect estimate (β = −2.07, *t* = −3.58). Dry cat food, mixed nuts and sake lees also tended to be depleted faster than the reference bait, although the effects were weaker and not statistically significant. The presence of raccoon dogs was associated with significantly longer bait depletion times (β = 1.37, *t* = 2.89), indicating slower overall bait removal when raccoon dogs visited the feeding stations, despite their limited direct access to bait due to the bottle‐shaped containers. Trial identity explained substantial variation in depletion time (random intercept SD = 1.30), suggesting strong among‐trial heterogeneity in overall bait depletion, consistent with site‐specific sink effects. Overall, these results indicate that bait performance is shaped not only by bait type, but also by interspecific interactions and local conditions at baiting sites.

**TABLE 2:** Linear mixed‐effects model results explaining variations in bait depletion time.

Predictor	Estimate	SE	*t*‐value
(Intercept)	9.684	0.623	15.56
Dry cat food	−1.057	0.575	−1.84
Tohato caramel corn	−2.072	0.578	−3.58
Japanese mixed nuts	−1.155	0.821	−1.41
Sake lees	−1.219	0.702	−1.74
Raccoon dog present (TRUE)	+1.369	0.473	2.89

*Note:* Response variables are presented as log‐transformed bait depletion times. A random intercept was included for each trial to account for between‐trial heterogeneity. Sample size: *n* = 122 observations in 46 trials. Random effects (standard deviation): Trial (intercept) = 1.296; Residual = 1.637. Intercepts represent expected depletion times (log scale) for the reference bait with raccoon dog presence = FALSE. Negative estimates are indicative of shorter depletion times (i.e., faster depletion), whereas positive estimates are indicative of longer depletion times (slower depletion). Statistical inferences (SE) are based on *t*‐values from the mixed model fit (lme4).

### 3.4 Long‐Term Space Use

Long‐term space‐use estimation via AKDE showed that bait stations were consistently embedded within the utilisation distributions of raccoons. All three bait stations were located within the 95% home‐range isopleth of all seven individuals, and for four of the seven individuals, the bait stations were also located within the 50% core‐area isopleth, indicating substantial individual variation in whether bait locations constituted core space (Table 3). Estimated 95% home‐range size varied from 0.98 to 16.72 km^2^ across individuals, while estimated 50% core‐area size ranged from 0.11 to 4.39 km^2^, indicating substantial heterogeneity in long‐term space use among raccoons in the post‐disaster agricultural landscape.

**TABLE 3:** Relationship between raccoon AKDE isopleths and bait stations showing long‐term space use.

Individual	Bait site	Within 50% core	Within 95% home range
Rac01F	Ogawara	No	Yes
Rac02M	Morotake	Yes	Yes
Rac03M	Morotake	Yes	Yes
Rac04M	Morotake	No	Yes
Rac05F	Morotake	No	Yes
Rac06M	Tsushima	Yes	Yes
Rac07F	Tsushima	Yes	Yes

*Note:* For each individual, autocorrelated kernel density estimation (AKDE) was used to estimate long‐term utilisation distributions. Home ranges and core areas were defined as the 95% and 50% AKDE isopleths, respectively. ‘Within 50% core area’ and ‘Within 95% home range’ indicate whether the bait station assigned to each individual was located inside the corresponding point‐estimate AKDE isopleth polygon.

### 3.5 Mid‐Term Proximity to Trap Sites

Daily minimum distances to trap sites decreased significantly during baiting periods (Gaussian generalised linear mixed model on log‐transformed distances; β = −0.54, SE = 0.12, *z* = −4.54, *p* < 0.001), showing an approximately 41% decline in expected minimum distance during baiting (exp[β] = 0.59). This finding indicated that raccoons approached trap locations more closely on baiting days. This baiting effect remained significant even when the site was included as an additional fixed effect (β = −0.55, SE = 0.12, *z* = −4.63, *p* < 0.001), suggesting consistent bait‐driven proximity responses across the study sites. Further analysis to determine whether proximity responses differed by sex using an interaction model revealed no significant baiting × sex interactions (likelihood ratio test, *p* = 0.50), although this result should be interpreted cautiously given the small sample size and uneven sex representation.

### 3.6 Short‐Term Movement Behaviour

**FIGURE 5:**
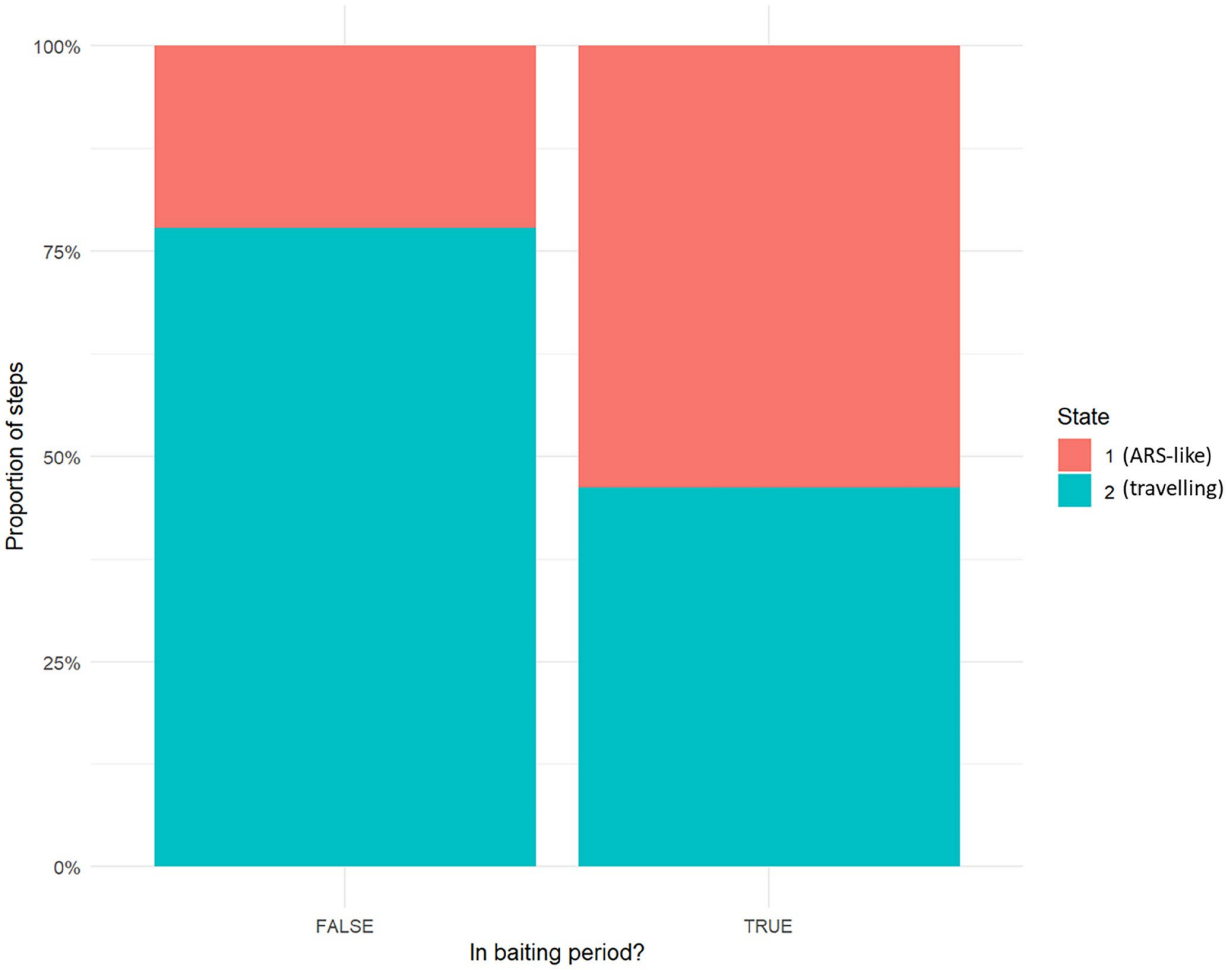
State composition (proportion of movement steps) estimated using hidden Markov models for high‐resolution GPS‐collared raccoons, with baiting and non‐baiting periods compared. State 1: Area‐restricted search (ARS)‐like behaviour; State 2: travelling behaviour.

HMMs fitted to high‐resolution individual data supported two distinct movement states with different step‐length and turning‐angle distributions. State 1 was characterised by short step lengths (mean ≈ 14 m) and low directional persistence, consistent with ARS‐like movements, whereas State 2 was characterised by much longer step lengths (mean ≈ 173 m) and higher directional persistence, consistent with travelling movements. State composition differed markedly between baiting and non‐baiting periods. Notably, pooling all high‐resolution individual data revealed that the proportion of steps assigned to ARS‐like State 1 increased from 22.2% during non‐baiting periods (*n* = 5,986) to 53.8% during baiting periods (*n* = 8,841; Figure 5), implying a strong short‐term behavioural response to bait deployment.

In the covariate‐dependent HMM, including baiting condition (bait_fac), distance to the nearest bait station (z_dist) and their interaction, state‐switching dynamics were consistent with localised attraction to bait stations. Further, during baiting periods, the probability of switching from travelling behaviour to ARS‐like behaviour (2 → 1) was the highest when individuals were close to bait stations (p21 = 0.121 at z_dist = −2), but it decreased as the distance to the bait station increased (p21 = 0.106 at z_dist = 2). Conversely, the probability of switching from ARS‐like behaviour back to travelling behaviour (1 → 2) was the lowest close to the bait stations during baiting periods (p12 = 0.088 at z_dist = −2) but increased with increasing distance to the bait station (p12 = 0.107 at z_dist = 2). Overall, baiting increased the likelihood that raccoons switched from travelling to ARS‐like behaviour and decreased the likelihood that they switched back from ARS‐like to travelling behaviour, especially when individuals were close to bait stations.

## 4 Discussion

### 4.1 Scale‐Dependent Responses to Baiting in a Human‐Abandoned Landscape

This study demonstrated that baiting induced scale‐dependent behavioural responses in invasive raccoons in a post‐disaster agricultural landscape. Using a cafeteria‐style feeding experiment combined with state‐based movement modelling (i.e., HMMs) and an intermediate‐scale proximity metric, we found convergent evidence that bait deployment generated immediate attraction near bait stations while also increasing daily proximity to trap locations. These findings support the view that baiting is not merely an operational tool for increasing trap visitation rates, but a management intervention that can influence fine‐scale movement decisions and local spatial concentration. However, the feeding experiment also showed substantial heterogeneity in first‐contact bait selection among individuals, indicating that attraction was not uniform across bait types or raccoons. Thus, baiting effectiveness likely depends not only on bait presence, but also on bait type and individual‐specific responsiveness. Although bait‐driven concentration processes can enhance management efficiency, they may also result in ecological risks if repeated aggregation occurs at predictable locations (Snow and VerCauteren 2019; Sorensen et al. 2014). More broadly, our findings highlight that movement‐informed approaches can improve the design and expected outcomes of bait‐based interventions, including wildlife vaccination strategies for raccoons, while also accounting for behavioural heterogeneity among target animals. Taken together, these results suggest that baiting can generate short‐term attraction; however, its management effectiveness will depend on context‐ and individual‐specific responses in complex post‐disaster landscapes.

### 4.2 Individual Heterogeneity in Bait Preference and Implications for Bait Selection

Raccoons exhibited pronounced individual heterogeneity in bait preference, although this pattern should be interpreted cautiously given the limited number of individuals. Rather than representing random noise around a single population‐level preference, these differences are consistent with broader evidence that individual‐level niche variation can be substantial even within populations of generalist consumers (Araújo et al. 2011; Bolnick et al. 2003). In raccoons and other omnivorous mesocarnivores, such heterogeneity is plausibly linked to behavioural flexibility in the use of spatially variable resources, and comparable patterns of marked interindividual variation in movement or resource use have been reported in raccoon dogs and coyotes occupying heterogeneous human‐modified landscapes (Daniels et al. 2019; Newsome et al. 2015; Saeki et al. 2007). Therefore, our results suggest that bait preference should be viewed both as a practical issue of trap optimisation and an expression of ecologically meaningful intraspecific variation. From a management perspective, this heterogeneity cautions against the assumption that a single ‘optimal bait’ will maximise population‐level effectiveness. While caramel corn and dry cat food were most frequently selected first overall, some individuals consistently preferred alternative baits such as sake lees, indicating that multi‐bait strategies may improve responsiveness in heterogeneous populations. More broadly, baiting outcomes are shaped not only by bait properties themselves but also by which individuals encounter, sample and repeatedly return to baited locations. This point is consistent with movement‐based studies of raccoons showing that variation in space use and encounter structure can influence bait uptake and management performance across landscapes (Beasley et al. 2015; Hill et al. 2024; McClure et al. 2022). Consequently, in human‐abandoned agricultural systems, where resource mosaics can be highly heterogeneous and foraging opportunities diverse, maintaining flexibility in bait composition may improve management performance while reducing overreliance on responses from only a subset of individuals.

### 4.3 Non‐Target Interference and Context Dependence of Bait Performance

Bait depletion dynamics varied by bait type and were influenced by non‐target activity at feeding stations, indicating that interspecific interactions can influence bait performance. Frequent visitation by raccoon dogs and other non‐target animals suggests that feeding stations functioned as multi‐species resource patches rather than raccoon‐only attractants. This created substantial opportunities for interference competition, altered access to bait resources and context‐dependent depletion dynamics. Such patterns are consistent with broader evidence that non‐target uptake and competitive interactions can reduce the efficiency and predictability of bait‐based management interventions (Snow and VerCauteren 2019; Sorensen et al. 2014). Our results further suggested that non‐target activity may not simply accelerate bait depletion via additive consumption, but also slow down depletion in some trials. In our system, this pattern was unlikely to reflect direct bait removal by raccoon dogs because the bait containers restricted access by non‐target species. Instead, the presence of raccoon dogs within confined feeding stations may have reduced uninterrupted feeding opportunities for raccoons by increasing caution, delaying access, or interrupting feeding bouts. Such a mechanism is consistent with interference competition and vigilance‐foraging trade‐offs whereby nearby competitors can reduce feeding efficiency even without directly consuming the focal resource (Vanak and Gompper 2010; Halliday and Morris 2013).

### 4.4 Short‐Term Behavioural Attraction: Baiting Shifts Movement States Near Bait Stations

HMMs provided clear evidence of short‐term behavioural attraction to bait stations. During baiting periods, raccoons spent a substantially greater proportion of time in an ARS‐like state and proximity to bait stations increased transitions into this state while reducing transitions out of it. These findings support the mechanism that baiting alters fine‐scale movement decisions by creating localised attractors that promote residency behaviour near management sites. Importantly, similar movement‐based mechanisms have been highlighted in raccoon oral rabies vaccination research, where bait encounters and uptake are influenced by habitat selection, space use and the spatial arrangement of baited areas relative to raccoon movements (Hill et al. 2024; McClure et al. 2022). Comparable conclusions have been reported for invasive wild pigs in studies using GPS‐based movement analyses to quantify bait‐site visitation and movement responses relevant to the design and expected performance of toxic baiting strategies (Lavelle et al. 2018). Operationally, increased residency near bait sites may enhance detectability and capture probability by increasing the time individuals spend in the vicinity of traps. However, the same mechanism can increase local contact opportunities among conspecifics and between target and non‐target species, reinforcing the need to design baiting strategies that balance capture efficiency with potential ecological and epidemiological risk mitigation (Snow and VerCauteren 2019; Sorensen et al. 2014).

### 4.5 Mid‐Term Proximity Effects: Daily Minimum Distance as an Operationally Relevant and Biologically Intermediate Signal

Beyond immediate behavioural state shifts, the reduction in daily minimum distance indicates that baiting increased day‐level proximity to trap sites in a manner that was not captured by either short‐term state classification or long‐term home‐range estimation alone. This result suggests that baiting effects operate at an intermediate temporal scale: raccoons did not simply show momentary attraction near bait sites, but also incorporated baited management locations more closely into their daily movements on baiting days. Simultaneously, the AKDE results showed little evidence of broader restructuring of long‐term space use, implying that increased daily proximity and long‐term redistribution are not equivalent responses. This distinction is scientifically important because it shows that baiting can intensify local use of management locations without necessarily altering the larger spatial structure of home ranges. Therefore, daily minimum distance is not only an operational metric; it also captures a biologically meaningful intermediate response that helps separate short‐term behavioural attraction from broad‐scale space‐use change.

### 4.6 Long‐Term Space Use: Bait Sites Overlapped With Established High‐Use Areas, but With Limited Inference Regarding Persistence

AKDE analyses indicated that bait stations were consistently located within the home ranges of the analysed individuals and, for four of the seven individuals, within the estimated 50% core areas. Because these analyses were restricted to raccoons that visited bait sites, this overlap likely reflects the space use of bait‐interacting individuals and should not be interpreted as representative of the full local raccoon population. These findings indicate that bait stations were embedded within the long‐term utilisation distributions of tracked raccoons, suggesting that baiting primarily intensified local use within existing home ranges rather than restructuring broader patterns of space use. However, interpreting these overlaps as evidence of sustained bait‐driven spatial dependence requires caution. Movement‐based studies of raccoon baiting interventions suggest that bait uptake and intervention effects can arise through repeated exposure and encounter processes mediated by movement and spatial access, without necessarily implying long‐term restructuring of home‐range boundaries (Beasley et al. 2015; Hill et al. 2024; McClure et al. 2022). In post‐disaster management settings, bait placement and subsequent trapping operations may also continue beyond formal trial periods, making it difficult to fully isolate bait‐induced persistence from ongoing intervention. Thus, our long‐term results are most appropriately interpreted as showing that bait deployment areas were embedded within established use areas. Within this spatial context, baiting generated strong short‐ and mid‐term responses, but did not provide clear evidence for home‐range boundary restructuring.

### 4.7 Implications for Invasive Mesocarnivore Management in Post‐Disaster Landscapes

The Fukushima evacuation zone presents a socio‐ecological system characterised by prolonged reductions in human activity, rapid landscape change and altered wildlife assemblages (Fukasawa et al. 2016; Lyons et al. 2020), conditions that can facilitate invasive species establishment by reducing management pressure and increasing the availability of refugia and resource (Didham et al. 2007; Hirose et al. 2021; Hobbs and Huenneke 1992). Our analysis of raccoon responses to baiting in this area demonstrated that baiting remains a powerful tool for eliciting behavioural attraction and increasing proximity to management sites even in post‐disaster and unmanaged landscapes. Simultaneously, this behavioural and increased proximity highlights the need for careful baiting design to balance operational benefits against potential ecological risks. Evidence from raccoon vaccination research suggests that baiting efficacy depends on the bait deployment strategy and habitat context and that movement‐informed approaches can substantially improve outcomes (Beasley et al. 2015; Hill et al. 2024; McClure et al. 2022). Therefore, baiting protocols that incorporate local habitat structure, individual heterogeneity in bait preference and account for the possibility of interspecific interference at feeding sites are of great significance in the management of various post‐disaster landscapes.

### 4.8 Limitations and Future Directions

Our inference was constrained by the limited number of GPS‐collared individuals that visited bait sites and by heterogeneity in fix intervals among individuals, which likely reduced the sensitivity of the HMM analyses to short‐duration state switching in low‐resolution tracks. Moreover, the small sample size and uneven sex representation limited the statistical power to detect sex‐specific responses; thus, the non‐significant baiting × sex interaction should be interpreted cautiously. In addition, because some analysed individuals were captured at or very close to bait stations, the strongest bait‐site effects may have been observed among raccoons already residing near those locations, rather than demonstrating attraction over broader spatial scales. This limitation is important when interpreting the high proportion of detections involving tagged raccoons and the long‐term overlap between bait stations and estimated space use. More broadly, the pronounced individual heterogeneity observed in bait preference and site use suggests that conclusions drawn from a small number of tagged individuals may not fully represent the range of bait‐response strategies present in the wider population. Similar challenges are recognised in raccoon baiting and oral rabies vaccination studies, where bait encounter, uptake and intervention outcomes depend strongly on movement behaviour, habitat use and spatial access to baited areas (Beasley et al. 2015; McClure et al. 2022; Hill et al. 2024). Simultaneously, the consistency between the HMM‐based behavioural responses and the daily minimum distance analyses suggests that the observed attraction to baited sites was not restricted to a single analytical framework or temporal scale. Therefore, future studies should aim to increase the sample size and strengthen causal inference by explicitly manipulating bait placement relative to established core areas or movement corridors, and by varying bait density and deployment duration. Such designs would help distinguish immediate attraction from longer‐term spatial dependence and clarify whether different behavioural types respond differently to the same baiting regime. Incorporating explicit measures of non‐target activity and station‐level competitive context would also improve inference, particularly in multi‐species systems where interference may alter feeding opportunities without direct bait removal. Together, these steps would help clarify when baiting changes only short‐term encounter processes and when it has stronger consequences for repeated site use or broader space‐use structure.

## 5 Conclusions

In this study, we examined the responses of raccoons to baiting as a control measure. By integrating bait preference experiments with GPS‐based movement analyses across behavioural and spatial scales, we demonstrated that baiting induces strong localised behavioural attraction and measurable mid‐term proximity responses to management sites in invasive raccoons in a post‐disaster agricultural landscape. While bait stations overlapped with established high‐use areas at longer time scales, inferring persistent bait‐driven spatial dependence was constrained by ongoing management activities. Overall, baiting can enhance management effectiveness while concentrating activity near predictable locations. Thus, improving control requires baiting protocols that account for individual heterogeneity in bait preference, non‐target interactions and scale‐dependent ecological consequences.

## Author Contributions

**Akira Watanabe:** conceptualization (equal), data curation (supporting), formal analysis (supporting), investigation (lead), methodology (equal), resources (lead), validation (supporting), visualization (supporting), writing – original draft (lead), writing – review and editing (equal). **Daisuke Nakamura:** conceptualization (lead), data curation (lead), formal analysis (lead), funding acquisition (supporting), investigation (supporting), methodology (lead), project administration (equal), resources (supporting), software (lead), supervision (supporting), validation (lead), visualization (lead), writing – original draft (supporting), writing – review and editing (lead). **Masataka Yoshida:** conceptualization (equal), formal analysis (supporting), funding acquisition (supporting), investigation (supporting), methodology (equal), project administration (supporting), resources (supporting), validation (supporting), writing – original draft (supporting), writing – review and editing (supporting). **Norihiro Hoshi:** conceptualization (equal), funding acquisition (lead), methodology (supporting), project administration (lead), resources (supporting), supervision (lead), writing – original draft (supporting), writing – review and editing (supporting).

## Funding

This work was supported by Agriculture, Forestry and Fisheries Research Council, JPFR24060105. Japan Society for the Promotion of Science, 25K08231.

## Conflicts of Interest

The authors declare no conflicts of interest.

## Data Availability

The data and code supporting the findings of this study are available from Dryad at https://doi.org/10.5061/dryad.3tx95x6wr.
